# Supplementary material for: Residual ANTXR1+ myofibroblasts after chemotherapy inhibit anti-tumor immunity via YAP1 signaling pathway
Source: Nat Commun. 2024 Feb 12;15:1312. doi: 10.1038/s41467-024-45595-3 (PMC10861537; doi:10.1038/s41467-024-45595-3)
Supplement: Supplementary file 1 — Supplementary Information [file 41467_2024_45595_MOESM1_ESM.pdf]

SUPPLEMENTARY INFORMATION

FOR

Residual ANTXR1+ myofibroblasts after chemotherapy inhibit anti-tumor immunity via YAP1 signaling pathway

Monika Licaj[1,2,#], Rana Mhaidly[1,2,#], Yann Kieffer[1,2,&], Hugo Croizer[1,2,&], Claire Bonneau[1,2,3,&], Arnaud Meng[1,2], Lounes Djerroudi[1,2,4], Kevin Mujangi-Ebeka[1,2], Hocine R. Hocine[1,2], Brigitte Bourachot[1,2], Ilaria Magagna[1,2], Renaud Leclere[4], Lea Guyonnet[5], Mylene Bohec[6], Coralie Guérin[5], Sylvain Baulande[6], Maud Kamal[7], Christophe Le Tourneau[7,8], Fabrice Lecuru[9], Véronique Becette[10], Roman Rouzier[3], Anne Vincent-Salomon[4], Geraldine Gentric[1,2,*] and Fatima Mechta-Grigoriou[1,2,*]

Co-Corresponding authors: Fatima Mechta-Grigoriou, Phone: +33 (0)1 56 24 66 53, E-mail address: fatima.mechta-grigoriou@curie.fr; Géraldine Gentric, Phone: +33 (0)1 56 24 66 45, E-mail address: geraldine.gentric@curie.fr

Supplementary Figure S1: Impact of CAF variations on HGSOC patient survival and gating strategy used for identification of CAF populations

(A) Kaplan Mayer curves showing the overall survival (OS, **Left**) and the disease-free survival (DFS, **Right**) of HGSOC platin-resistant patients showing enrichment either in myofibroblasts (CAF-S1 and CAF-S4) (N = 7, black curve) or in normal-like fibroblasts (CAF-S2 and CAF-S3) (N = 7, grey curve) after chemotherapy. Log-rank test. **(B)** Multivariate Cox regression analysis for OS (**Left**) and DFS (**Right**) considering enrichment of CAF populations and residual epithelium content after chemotherapy. **(C)** Flow cytometry plots showing the gating strategy to identify CAF subsets in treatment-naïve (**Up**) and after chemotherapy (**Bottom**) HGSOC samples. Cells are gated on Live/Dead$^-$, EPCAM$^-$, CD45$^-$, CD31$^-$ and CD235a$^-$ to exclude dead, epithelial, hematopoietic, endothelial cells and erythrocytes, respectively. % of all gated populations are indicated.

Retrospective Curie 1 cohort

A

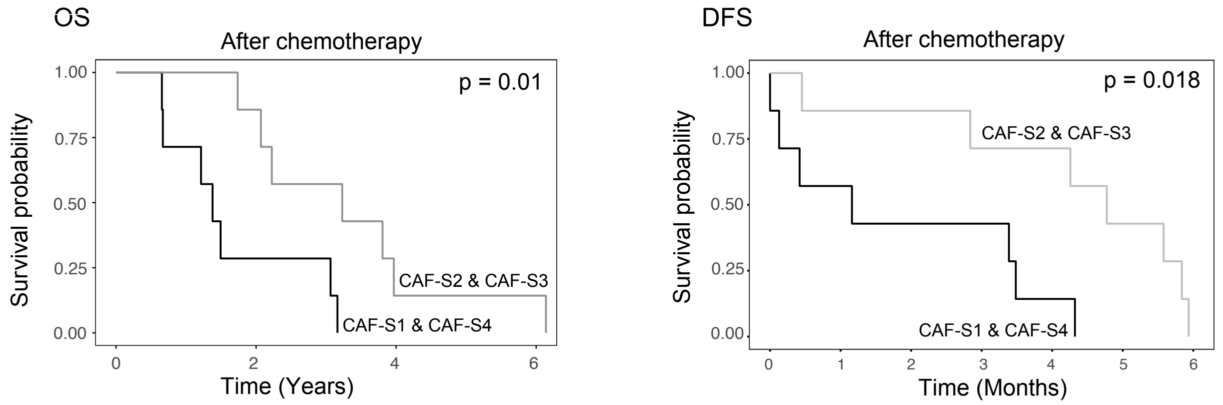

B

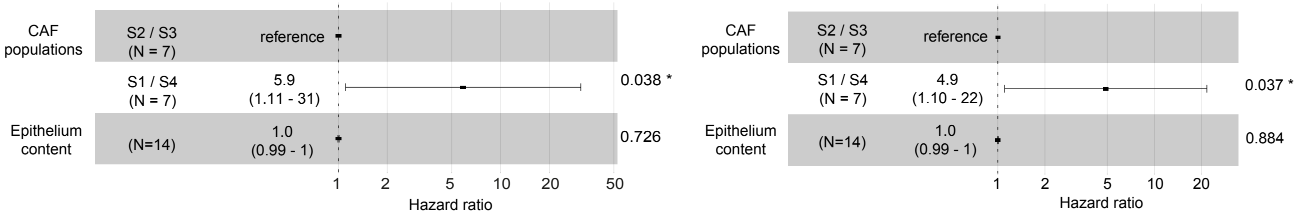

Prospective cohort 1

C

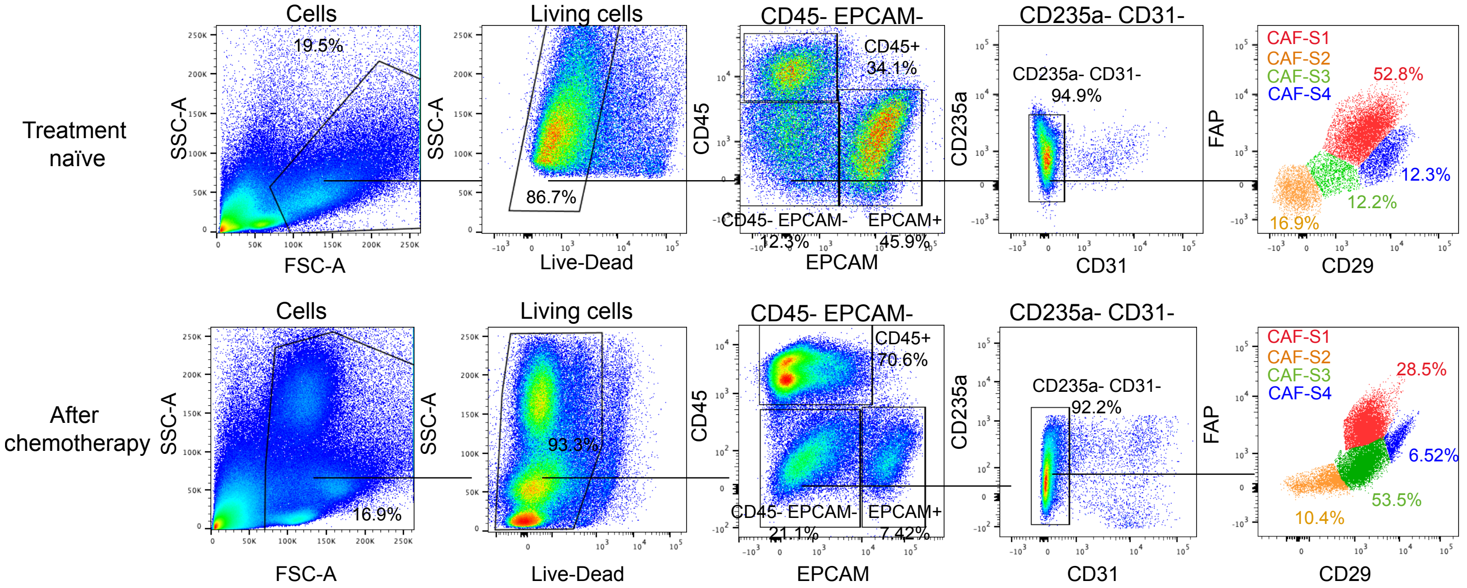

Supplementary Figure S1

Supplementary Figure S2: Correlations between content in CAF populations and TILs in HGSOC

(**A**) Number of FOXP3$^+$ T lymphocytes per mm^2 in HGSOC stromal and epithelial compartments, respectively, before and after chemotherapy. Data are mean ± SEM (N = 35). P-values from Wilcoxon paired test. **(B)** Number of FOXP3$^+$ TILs per mm^2 normalized to the stromal and epithelial content per sample. Data are mean ± SEM (N = 35). Two-sided Wilcoxon paired test. (**C, D, E**) Variations of CAF-S1, CAF-S4 and TILs upon chemotherapy, as shown in (**Figure 2E**). Each dot represents one tumor (N = 35). Two-sided Spearman correlation test. In (**C**), scatter plots between the variations in the content of CAF-S1 assessed by FAP H-score) and the number of CD3$^+$ (**Left**), CD8$^+$ (**Middle**) or FOXP3$^+$ (**Right**) TILs per mm^2 of total section. (**D**) Same as in (**C**) showing the variation in the content of both CAF-S1 and CAF-S4 myofibroblasts (assessed by SMA H-score). (**E**) Same as in (**D**) for both CAF-S1 and CAF-S4 myofibroblast content (assessed by the delta SMA H-score, ΔH-score SMA). (**F**) Correlations plots with linear regression lines between % of CD8$^+$ among CD3$^+$ TILs and % of CAF-S4 among CAF before chemotherapy (treatment-naïve, **Left**), after chemotherapy (**Middle**) and before and after chemotherapy (Circles: treatment-naïve; Triangles: after chemotherapy) (**Right**). Data are from flow cytometry (Prospective cohort 1). N = 16 patients. Two-sided Spearman correlation test.

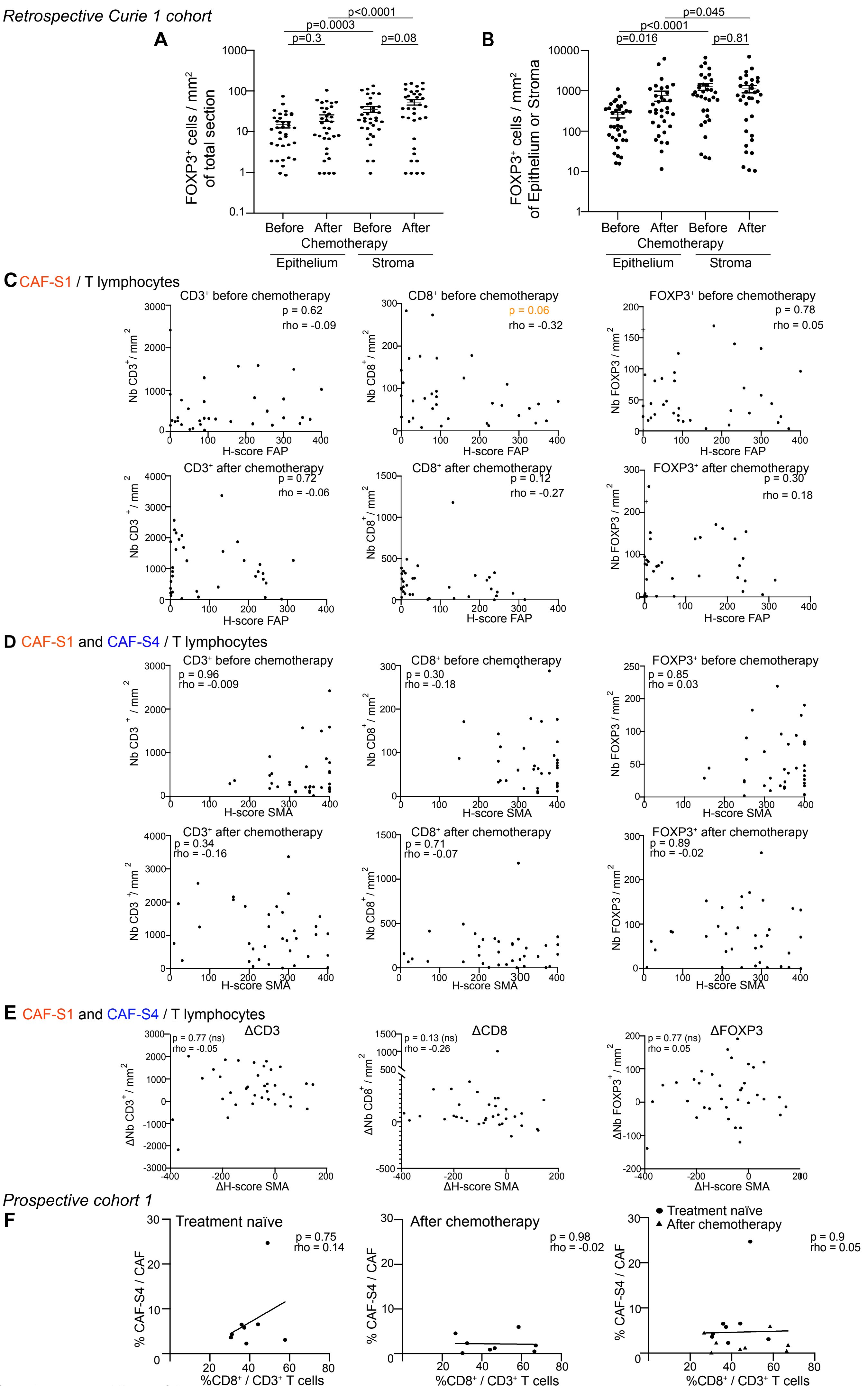

Supplementary Figure S2

Supplementary Figure S3: Annotation of CAF-S1 clusters in scRNAseq datasets from HGSOC and BC

(**A-C**) UMAP showing patient distribution (**Top**) and prediction scores (**Bottom**) of CAF-S1 clusters assessed by label transfer in scRNAseq from HGSOC Curie cohort (N = 12 patients: 5 treatment naïve; 7 treated) (**A**), HGSOC Turku cohort (N = 11 paired treatment naïve and treated patients) (**B**) and BC cohort (N = 42 patients: 31 treatment naïve; 11 treated) (**C**). (**D**) Heatmap showing the relative contribution of the 10 factors obtained after consensus Non-Negative Matrix factorization (cNMF) of CAF-S1 fibroblasts from HGSOC Curie cohort (Prospective cohort 2, N = 12 patients: 5 treatment naïve; 7 treated). Clustering uses correlation distance and Ward D2 agglomeration method. Bars on top of the heatmap represent CAF-S1 cluster identity (colors) and treatment status (grey/black). Representative biological pathways from the top 200 most informative genes of factors 1, 2, 3 and 6 are indicated on the right side for the most abundant CAF-S1 clusters analyzed in this study. (**E)** UMAP of 7 959 CAF-S1 from the BC cohort (N=42 patients including 31 treatment-naive and 11 after chemotherapy) colored by treatment status (**Left**) or by CAF-S1 clusters predicted using label transfer (**Middle**). **Right**, Proportion of CAF-S1 clusters among CAF-S1. Two-sided Fisher's exact test. **(F-H**) Impact of down-sampling of CAF-S1 in scRNAseq data from the BC cohort (N = 42 patients). (**F**) UMAP showing CAF-S1 cluster distribution predicted by label transfer. (**G**) ANTXR1 expression (**Left**) and % of ANTXR1$^+$ CAF-S1 among total CAF-S1 (**Right**) in treatment-naïve patients (N = 31) and after chemotherapy (N = 11). Two-sided Wilcoxon (**Left**) and Fisher's Exact test (**Right**). (**H**) Proportion of CAF-S1 clusters among CAF-S1 in treatment-naïve and chemotherapy-treated samples. Two-sided Fisher's Exact test.

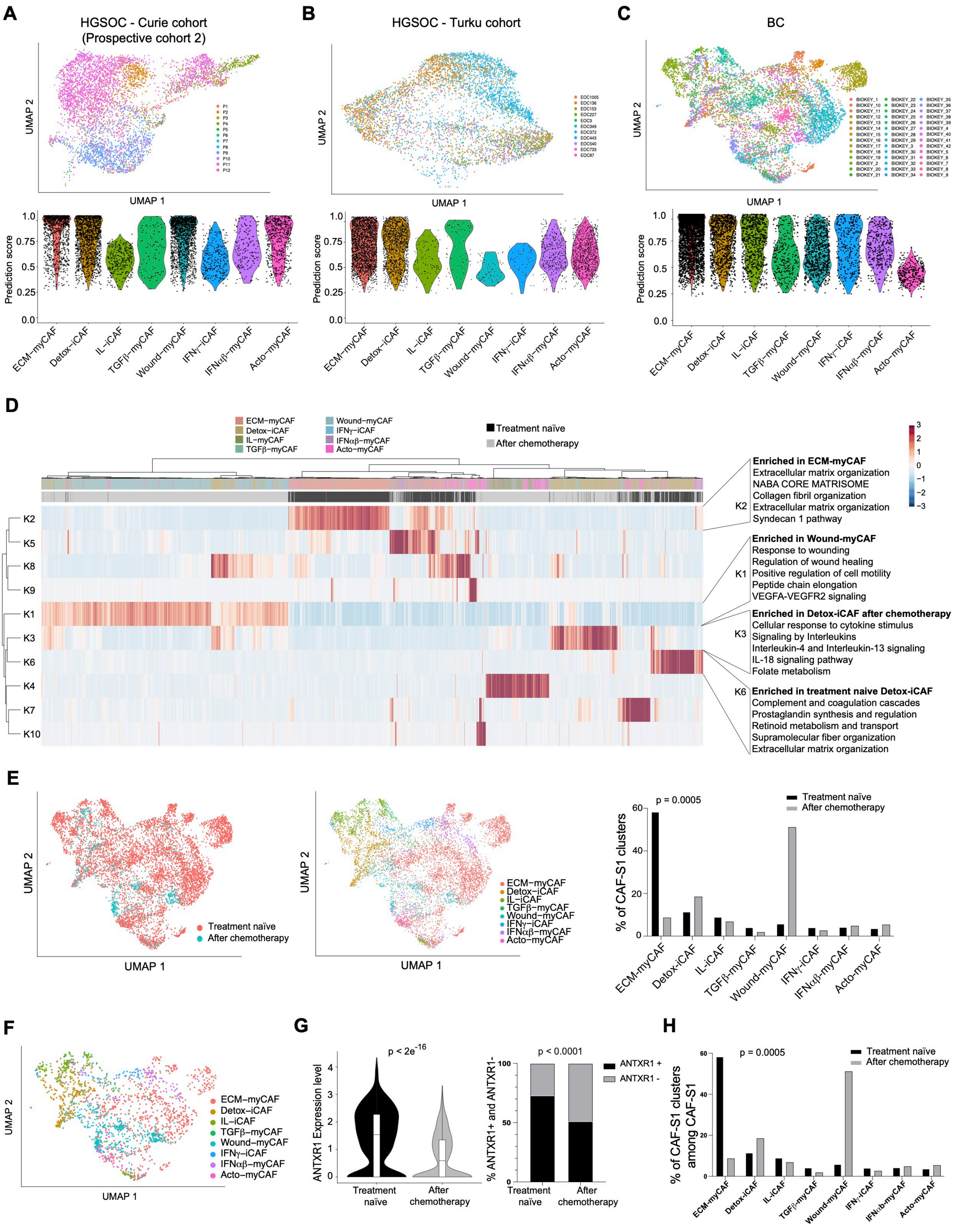

Supplementary Figure S3

Supplementary Figure S4: HGSOC cellular atlas for deconvolution of bulk RNAseq and spatial data

(**A**) UMAP of 49 909 cells from HGSOC, which encompass 24 different cell types and states and compose a comprehensive HGSOC cellular atlas. (**B**) Expression values of specific genes in the 24 cell types and states of the HGSOC atlas. Colors highlight average expression of each gene across all cells within a cell type. Circle sizes represent the % of cells expressing a given gene. (**C**) **Left**, Proportion of cancer cells among total cells in the retrospective SCANDARE Curie 2 cohort before (treatment-naïve) and after chemotherapy. (N=70 HGSOC including 45 treatment-naïve, 25 after chemotherapy). Two-sided Mann-Whitney test. **Right,** Same as in **(Left)** according to response to chemotherapy. Two-sided Mann-Whitney test. (**D**) **Top**, H&E staining of the 10 spatial transcriptomic sections analyzed at baseline (**Left**, Treatment-naïve) and in residual disease (**Right**, After chemotherapy). **Bottom**, Pathological annotations of the sections distinguishing spots covering the epithelial compartment (red) and the stromal compartment (blue). (**E**) % of stroma (determined by morphological analysis on HES staining) in HGSOC sections analyzed by spatial transcriptomics. Two-sided Welch t-test. (**F**) Number of $CD3^+$ (**Left**) and $CD8^+$ (**Right**) T lymphocytes identified per spot after deconvolution in the spatial transcriptomic data before and after treatment. At least one $CD3^+$ T lymphocyte per spot is identified by deconvolution in 16 194 spots out of 37 643 spots (average abundance of $CD3^+$ or $CD8^+$ per spot = 2.16 ($CD3^+$) and 0.52 ($CD8^+$); average abundance of all cells per spot = 10.2). n = 37 643 total spots (13 438 Treatment naïve; 24 205 after chemotherapy. Two-sided Mann-Whitney test.

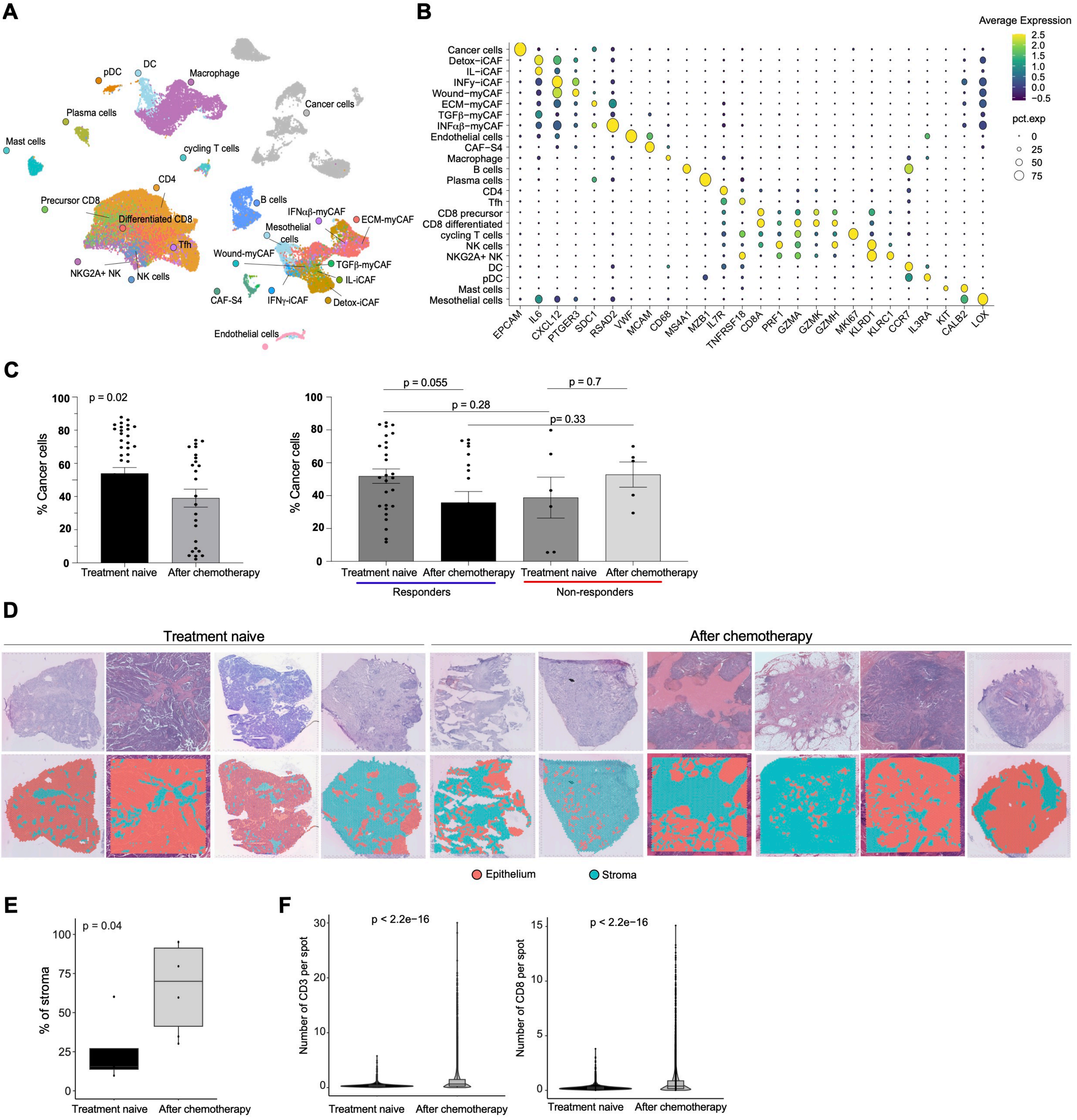

Supplementary Figure S4

Supplementary Figure S5: Validity tests for functional assays

(**A**) Expression of TEAD-target genes in ECM-myCAF compared to all other CAF-S1 clusters. Data are from scRNAseq (n = 5 618). Two-sided Mann-Whitney test. (**B**) YAP1 staining on sections from paraffin-embedded CAF-S1 cell line (Hs578T) after transfection with siCtrl or siYAP1(1)/siYAP1(2). Scale bar = 50μm. (n = 5). (**C**) **Left**, Western blot showing YAP1 protein level from Hs578T CAF-S1 cell line transfected with siCtrl or with specific siRNA against YAP1 (siYAP1(1), siYAP1(2)). Actin is used as an internal control for protein loading. **Right**, Corresponding quantifications of YAP1 protein levels normalized to Actin (n = 6). (**D**) Correlation plots between YAP1 H-score in the stroma and SMA H-score in HGSOC before (**Left**) and after chemotherapy (**Right**). Data are from IHC staining (N = 35, Retrospective Curie 1 cohort). Two-sided Spearman test. (**E**) Correlation plots between the % of CAF with nuclear $YAP1^+$ in the stroma and SMA H-score in HGSOC before (**Left**) and after chemotherapy (**Right**). Data are from IHC staining (N = 35, Retrospective Curie 1 cohort). Two-sided Spearman test.

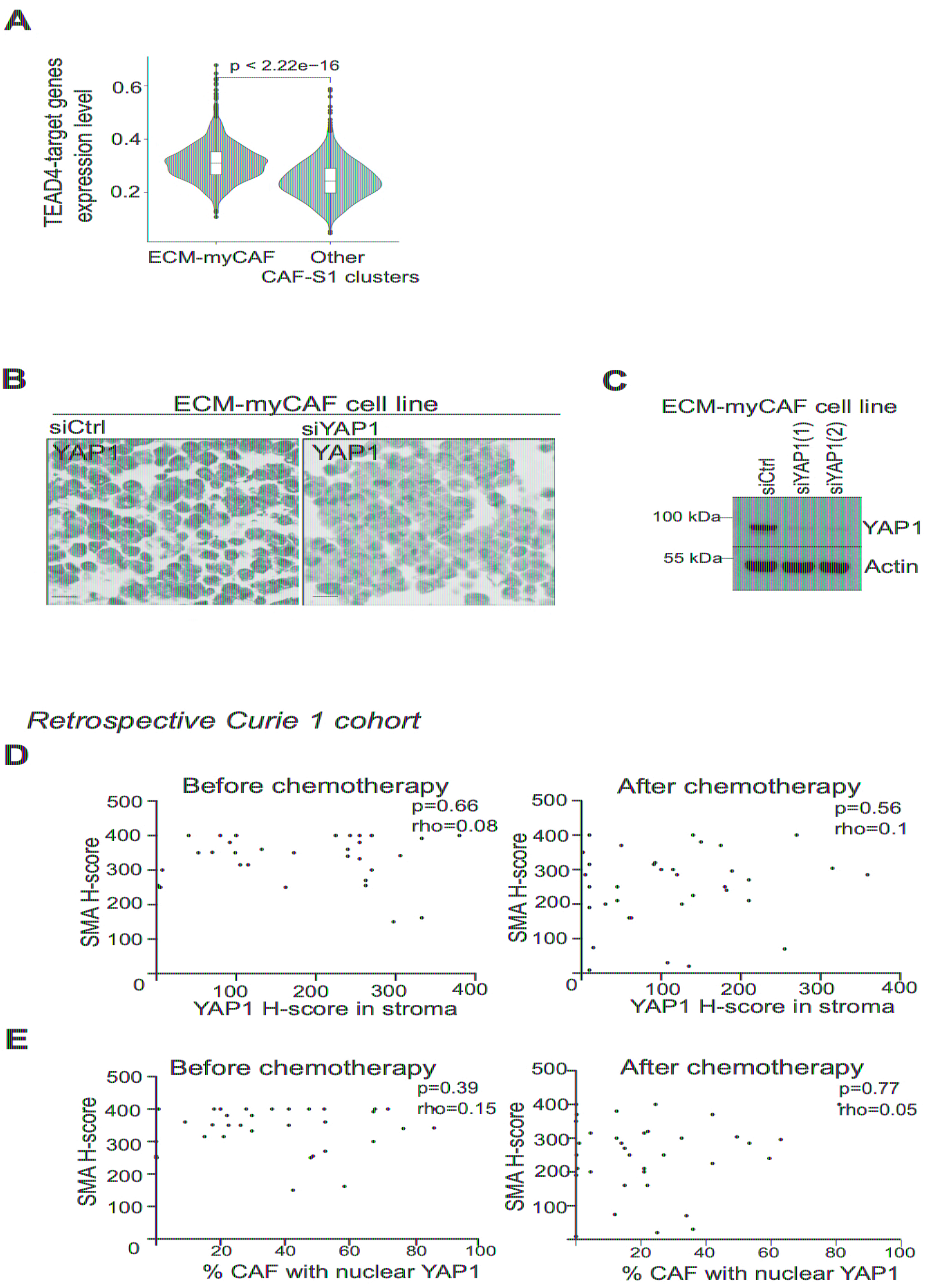

Supplementary Figure S5

Supplementary Figure S6: Characterization of Detox-iCAF and ECM-myCAF

(**A**) Gating strategy used to characterize primary ECM-myCAF (**Up**) and Detox-iCAF (**Middle**) isolated from HGSOC samples. CAF-S1 cells are gated on FAP^{+} $CD29^{+}$ fibroblasts. Among CAF-S1 cells, ANTXR1 protein levels is used to distinguish $ANTXR1^{+}$ myofibroblastic ECM-myCAF from $ANTXR1^{-}$ inflammatory Detox-iCAF. (**Bottom**) Gating strategy used to characterize $CD8^{+}$ T lymphocytes among total $CD3^{+}$ T cells. (B) Heatmap showing scaled expression of the 233 genes from the NABA matrisome signature (**Table S3**), which are up-regulated in ECM-myCAF cultured in plastic dishes (n = 3 primary CAF-S1 cell lines), and the 25 genes from the inflammatory signature (**Table S4**), which are up-regulated in Detox-iCAF cultured on collagen-coated dishes (n = 3 primary CAF-S1 cell lines). (**C**) Boxplots showing the normalized and log2-transformed expression of 8 representative genes from the NABA matrisome signature in ECM-myCAF (plastic-condition, n = 3) *versus* Detox-iCAF (collagen-condition, n = 3). Wald test using DESeq2 after correction using Benjamini and Hochberg method. (**D**) Same as (**C**) for 8 representative genes from the inflammatory signature.

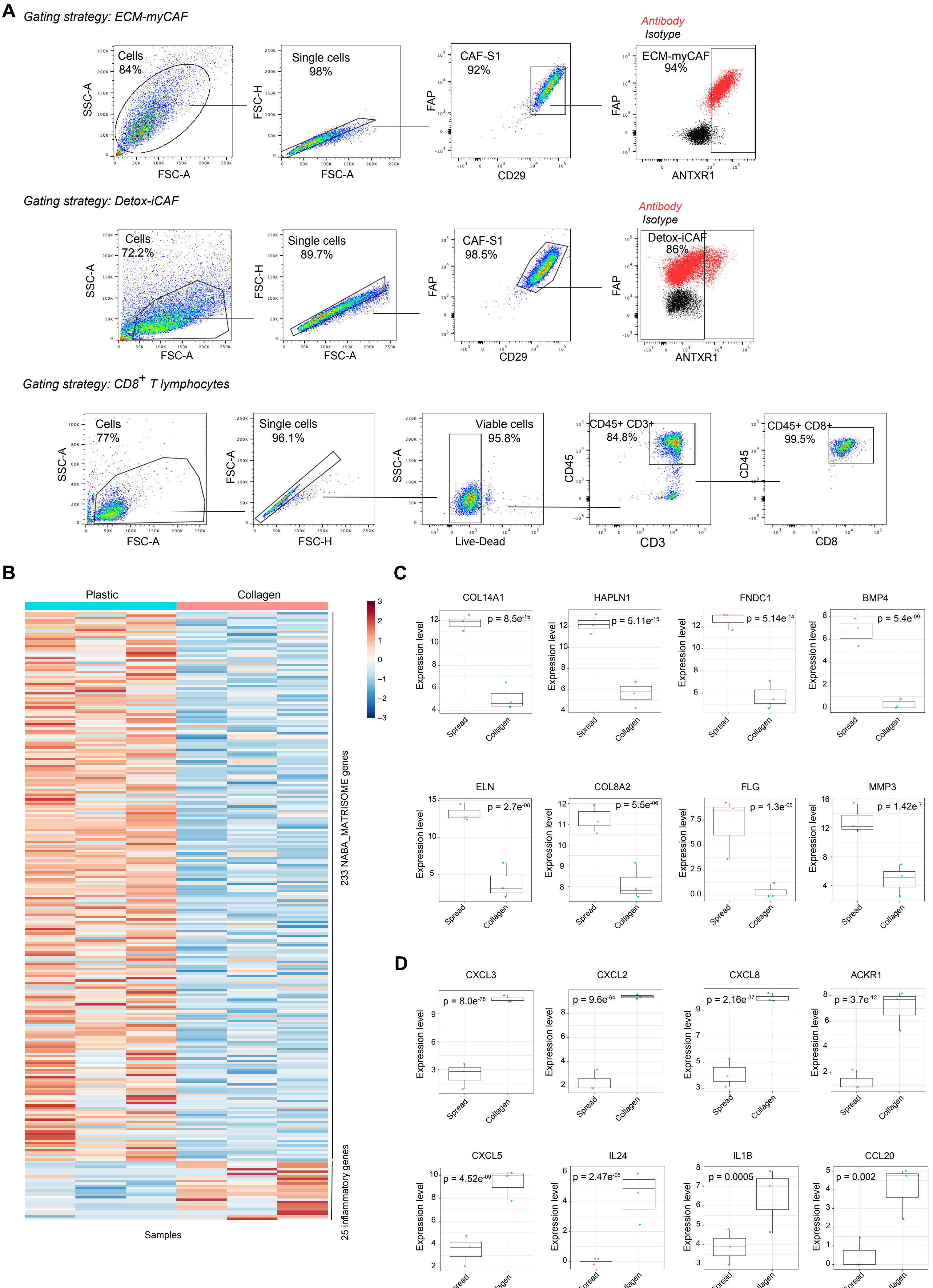

Supplementary Figure S6

Supplementary Figure S7: Functional assays comparing Detox-iCAF and ECM-myCAF properties

(**A**) Data are same as in (**Fig. 6 A-D**) but here analyzed using unpaired statistical tests. **Up Left**, Bar plots showing the % of PD-1^+ T cells among $CD8^+$ T lymphocytes (**Left**) and the specific mean fluorescence intensity (speMFI) of PD-1 among PD-1^+ $CD8^+$ T cells (**Right**). Data are mean ± SEM (n = 5). Two-sided Mann-Whitney test. **Up Right** and **Bottom**: Same as (**Up Left**) for Granzyme B, Perforin and IFN-γ . Data are mean ± SEM (n = 5). Two-sided Student t-test. (**B**) **Left,** Western blot showing YAP1 protein levels in human primary ECM-myCAF and Detox-iCAF fibroblasts transfected either with non-targeted siRNA (siCtrl) or with two specific siRNA against *YAP1* (siYAP1(1), siYAP1(2)). Actin is used as an internal control for protein loading (n = 5). (**C**) **Left**, *CYR61* expression in CAF-S1 compared to CAF-S4 fibroblasts from RNAseq data of untreated HGSOC samples (n = 8 CAF-S1 and n = 6 CAF-S4). Two-sided Mann Whitney test. **Right**, *CYR61* expression in CAF-S1 cells isolated from HGSOC patients (N = 8) compared to primary ECM-myCAF cultured in plastic dishes (n = 3). Data are from RNAseq. Two-paired t-test. (**D**) Western blot showing CYR61 protein levels in human primary ECM-myCAF fibroblasts transfected either with non-targeted siRNA (siCtrl) or with specific siRNA against *CYR61* (siCYR61(1), siCYR61(2)). Actin is used as an internal control for protein loading (n = 6). (**E**) **From Left to Right**, % of Granzyme B^+ $CD8^+$, the % of $Perforin^+$ $CD8^+$, the % IFN-γ^+ $CD8^+$ and the % of PD-1^+ $CD8^+$ among total CD8+ T lymphocytes after 24h of co-culture with primary Detox-iCAF or ECM-myCAF transfected either with untargeted siRNA (siCtrl) or with two different siRNA targeting CYR61 (siCYR61(1), siCYR61(2)). Data are mean ± SEM (n = 6). Two-sided Wilcoxon test. (**F**) % of cancer cell death after 24h of incubation with $CD8^+$ T cells pre-incubated with primary Detox-iCAF or ECM-myCAF transfected either with untargeted siRNA (siCtrl) or with two different siRNA targeting CYR61 (siCYR61(1), siCYR61(2)). Data are mean ± SEM (n = 6). Two-sided unpaired Student t-test.

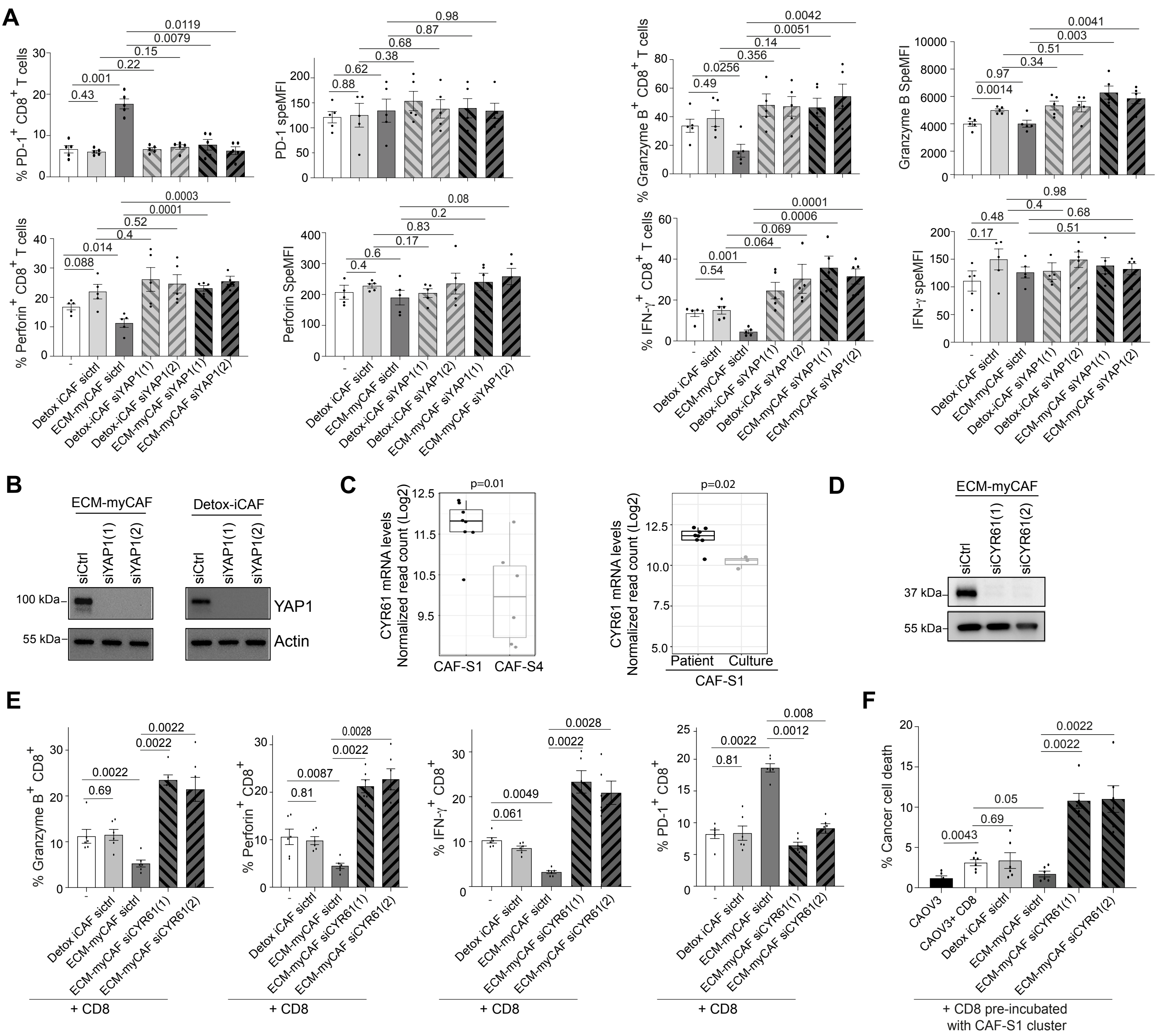

Supplementary Figure S7

Supplementary Table S1: List of antibodies used in this study

List of primary antibodies used for immunohistochemistry, flow cytometry analysis, cell sorting and western blot. Each antibody was titrated separately to find the right concentration to use. Appropriate isotype controls corresponding to the matched antibody were also used.

Immunohistochemistry (IHC)			
**Antibodies**	**Reference**	**Dilution**	**Antigen retrieval**
FAP-Rat IgG2a	Vitatex, #MABS1001	1/150	Citrate pH6
CD29-Mouse IgG1	Abcam, #ab3167	1/150	Citrate pH6
SMA-Mouse IgG1	Dako, #M0851	1/350	Citrate pH6
FSP1-Rabbit IgG	Abcam, #ab27957	1/450	Citrate pH6
YAP1-Rabbit IgG	Cell Signaling, #14074	1/800	EDTA pH9
EPCAM-Mouse IgG1	Dako, #M0804	1/200	Citrate pH6
CD3-Mouse IgG1	DAKO, #M7254	1/90	Citrate pH6
CD8-Mouse IgG1	DAKO, #M7103	1/100	Citrate pH6
FOXP3-Mouse IgG1	Abcam, #ab20034	1/100	EDTA pH9
ANTXR1-Rabbit IgG	Abcam #ab241067	1/400	pH9
**IgG controls**	**Reference**	**Dilution**	**Antigen retrieval**
Rabbit IgG-Isotype control for YAP1	Abcam, #ab171870	1/800	EDTA pH9
Rat IgG2a-Isotype control for FAP	Affymetrix, #14-4321	1/150	Citrate pH6
Mouse IgG1-Isotype control for SMA	Abcam, #ab91353	1/350	Citrate pH6
Mouse IgG1-Isotype control for CD29	Abcam, #ab91353	1/150	Citrate pH6
Mouse IgG1-Isotype control for FOXP3	Abcam, #ab91353	1/100	EDTA pH9
Mouse IgG1-Isotype control for CD3	Dako, #X093101	1/90	Citrate pH6
Mouse IgG1-Isotype control for CD8	Dako, #X093101	1/100	Citrate pH6
Mouse IgG1-Isotype control for EPCAM	Dako, #X093101	1/200	Citrate pH6
Rabbit IgG-Isotype Control for FSP1	Dako, #X090302	1/450	Citrate pH6

Multiplex			
**Antibodies**	**Reference**	**Dilution**	**Antigen retrieval**
ANTXR1-Rabbit IgG	Abcam #ab246321	5ug/ml	pH9
CD8 (AMC908)	RareCyte, #52-1048-501	0.5ug/ml	pH9
panCK (AE1/AE3/C11)	RareCyte, #52-1015-801	2.5ug/ml	pH9

Flow Cytometry (FACS)		
**Antibody pool for CAF subset characterization from HGSOC**	**Reference**	**Dilution**
Brilliant Violet 605-CD326 (EpCAM)	BioLegend, #324224	1/50
PE/Cy7-CD31	BioLegend, #303118	1/100
APC/Cy7-CD45	BD Biosciences, #BD-557833	1/20
PerCP5,5-CD235a	Biolegend, #349110	1/50
Unconjugated-FAP	R&D Systems, #MAB3715	1/100
Alexa700-CD29	BioLegend, #303020	1/100
Alexa594-αSMA	R&D Systems, #IC1420T-025	1/25
PE anti human S100A4 (FSP1)	BioLegend, # 370004	1/25
Fluorescent dye Zenon APC Mouse IgG1 labeling kit	Thermo Fisher Scientific, #Z25051	1/100
**IgG controls**	**Reference**	**Dilution**
Mouse IgG1 isotype control FAP	R&D Systems, #MAB002	1/200
Alexa Fluor® 700 Mouse IgG1, κ- Isotype control CD29	BioLegend, #400144	1/25
Mouse IgG2A Alexa Fluor 594-conjugated-Isotype control αSMA	R&D Systems, #IC003T	1/25
PE Mouse IgG1, κ- Isotype control FSP1	BioLegend, #400140	1/20
**Antibody pool for T lymphocytes characterization from HGSOC**	**Reference**	**Dilution**
APC, human-CD4	Miltenyi Biotec #130-113-210	1/20
APC-Cy7-CD45	BD Biosciences, #557833	1/20
Alexa Fluor 700-CD3	BD Biosciences, #557943	1/40
PE Alexa 610-CD8	Thermofisher, #MHCD0822	1/80
Live/Dead Fixable Violet Dead Cell Stain Kit, for 405 nm excitation	Thermofisher, #L34955	1/1000
**Antibody pool for CAF-S1 primary cell characterization**	**Reference**	**Dilution**
Unconjugated-FAP	R&D Systems, #MAB3715	1/100
Alexa700-CD29	BioLegend, #303020	1/100
Mouse anti TEM8/ANTXR1 AF405	Novus Biologicals, #NB100-56585AF405	1/25
**IgG controls**	**Reference**	**Dilution**
Mouse IgG1 isotype control FAP	R&D Systems, #MAB002	1/200
Alexa Fluor® 700 Mouse IgG1, κ- Isotype control CD29	BioLegend, #400144	1/25
Mouse IgG1 Isotype Control AF405	Novus Biologicals, #IC003T	1/25
**Antibody pool for CAF-S1 cell sorting**	**Reference**	**Dilution**
PE anti human EPCAM	Biolegend #324205	1/100
PEcy7 anti human CD31	Biolegend #303118	1/100
APCcy7 anti human CD45	BD Biosciences #557833	1/25
FITC anti human CD235a	Biolegend #349104	1/50
Unconjugated-FAP	R&D Systems, #MAB3715	1/100
Alexa700-CD29	BioLegend, #303020	1/100
**IgG controls**	**Reference**	**Dilution**
Mouse IgG1 isotype control FAP	R&D Systems, #MAB002	1/200
Alexa Fluor® 700 Mouse IgG1, κ- Isotype control CD29	BioLegend, #400144	1/25
**Antibody pool for CD8+ cell solation for functional assays**	**Reference**	**Dilution**
Alexa Fluor 700-CD3	BD Biosciences, #557943	1/40
BV510 Mouse Anti-Human CD8	BD Biosciences, #563919	1/80
Alexa Fluor® 488 Mouse Anti-Human Perforin	BD Biosciences, #563764	1/25
PE Mouse Anti-Human Granzyme B	BD Biosciences, #561142	1/25
BV421 Mouse Anti-Human CD279 (PD-1)	BD Biosciences, #562516	1/20
BV786 Mouse ANti-Human IFN-g	BD Biosciences #563731	1/50
Fixable viability stain 780	BD Bioscience #565388	1/1000
**IgG controls**	**Reference**	**Dilution**
Alexa Fluor® 488 Mouse IgG2b, κ Isotype Control Perforin	BD Biosciences, #558716	1/25
PE Mouse IgG1, κ Isotype Control Granzyme B	BD Biosciences, #555749	1/25
BV421 Mouse IgG1, k Isotype Control PD-1	BD Biosciences, #562438	1/20
BV786 Mouse IgG1, k Isotype Control IFN-g	BD Biosciences #563330	1/50

Western Blot		
**Antibodies**	**Reference**	**Dilution**
YAP (D8H1X)	Cell Signaling #14074	1/1000
CYR61/CCN1	Novusbio #NB100-356	1/1000
Actin	Sigma #A5441	1/10.000

Supplementary Table S1

Supplementary Table S2: List of TEAD-target genes (based on Regulons) from the DoRothEA database

TEAD1_target genes		TEAD2_target genes	TEAD4_target genes	
MSLN	MACF1	CCND2	ABLIM2	TCF4
ADGRL2	MAML2	RIN3	ADAMTSL1	TGFBI
AMOTL2	MBNL1	AJUBA	ADGRL2	TGM2
GTDC1	MBNL2	ASAP3	ALK	TIAM2
ITGA1	MICAL2	ATP6V1A	ANXA2	TJP2
OSBPL9	MYLK	ATP8B2	BCAR3	TLE4
SORBS2	NAIP	CFI	BCR	TRIM2
TACC1	NAV2	CTDSP2	CCN2	VMP1
AFAP1	NDST1	CYBRD1	CEMIP2	ZMYND8
AHNAK	NEK7	DHRSX	CMIP	
ANK3	NRP2	DLGAP4	DAB2IP	
ANO6	NRXN3	DNAJB5	DENND1A	
APBB2	PALLD	DTNBP1	DENND2B	
ARHGAP24	PAM	FAM89A	DGKB	
ARHGAP29	PARD3B	GALNT2	DLGAP1	
ARHGAP35	PBX1	JMJD1C	ETV6	
ARNT2	PCYT1A	LAMB2	EXT1	
ASAP1	PDE4B	LSM10	FGFR2	
ASAP2	PDE4D	MAP1S	FNDC3B	
BCAR3	PDGFC	MPZ	FOXO3	
CACNA2D1	PDLIM5	MYL12A	GALNT2	
CALD1	PRICKLE2	PLEKHA4	GLIS3	
CAMK2D	PRKAG2	RHBDF1	GMDS	
CAPN2	PRKCH	SNRPC	GTDC1	
CAST	PRUNE2	SPEG	HDAC9	
CLIC5	PTPN14	ST3GAL2	HIVEP3	
CLIP4	PTPRM	TBC1D25	HSPG2	
CNOT1	RAPGEF2	TRAF4	IL1R1	
CRIM1	RARB	TRIP6	IQSEC1	
CTNNA1	RASAL2	VPS37B	ITPR1	
DAAM1	RB1CC1		KAZN	
DENND2B	RBMS1		KDM4C	
DGKB	RBMS3		KRT18	
DLC1	RERE		LITAF	
DLGAP1	RTN4		MACF1	
DNMBP	SAMD4A		MAML2	
DST	SASH1		MAP4K4	
EGFR	SCHIP1		MID1	
EML1	SEMA3C		MPZL1	
EPS8	SEMA3D		MYH9	
EXT1	SH3BP4		NEK6	
FAM160A1	SHROOM3		NIBAN2	
FAT1	SMYD3		PDE4B	
FEZ2	SORBS1		PDGFC	
FHL2	SORCS2		PDLIM1	
FLNB	SPIDR		PHLDB2	
FMN2	SPTBN1		PHLPP1	
FRMD6	STARD13		PICALM	
FTO	SVIL		PLCL1	
GMDS	TANC1		PLXNA2	
GULP1	TCF4		PRKCH	
HDAC9	TENM3		PTPRJ	
INPP4B	TGFB2		RABGAP1L	
ITGB1	TGFBR3		RAI14	
ITSN1	TIAM2		RBPMS	
JCAD	TLN2		RIN3	
KCNMA1	TMEM245		RIPOR3	
KDM4C	TRIO		RND3	
KIF13A	UACA		SIK3	
KTN1	UBE2H		SORBS2	
LAMC1	VPS13D		SPIDR	
LAMC2	WWC2		SPTB	
LIMCH1	WWTR1		STK10	
LPP	YAP1		TACC2	

Supplementary Table S2

Supplementary Table S3: List of genes from the NABA matrisome up-regulated in ECM-myCAF

List of the 233 genes from the NABA matrisome signature, which are up-regulated in ECM-myCAF cultured in plastic-dishes compared to Detox-iCAF maintained in collagen-coated dishes.

NABA_Matrisome_Signature Up-regulated in Plastic condition				
THBS3	HAPLN3	HTRA4	TNFSF13	CHRDL1
SPON1	PODNL1	FRZB	CLEC2L	IGFBP3
LUM	PRSS12	ADAM12	SERPINB10	ADAMTSL3
SEMA6A	LAMC3	S100A3	SULF2	FST
ADAMTS7	IL34	PLXDC1	C1QTNF3	FGF7
SEMA4D	TINAGL1	SCUBE1	ZPLD1	FGF16
SDC2	ANXA8	IGFBP2	EMID1	TNXB
CRISPLD1	DPT	PRELP	TGM5	ANXA13
ECM2	PRSS3	SERPINA9	BMP6	TNFSF15
EGFLAM	CRIM1	SVEP1	TGFB2	EMCN
ANGPT1	IGSF10	IL7	EFEMP1	PODN
C1QTNF7	TLL2	MMP10	SERPINB9	RSPO3
IGFBP6	ADAM22	NTNG2	IGFBPL1	SEMA3D
CLEC1A	IL17B	IGFALS	GDF7	SFRP4
CILP2	FGF1	EBI3	FGFBP3	MGP
VWF	VWA5A	F13A1	ANXA3	ADAMTS15
COL9A2	S100A4	SERPINA3	MUC1	RELN
IL12A	BCAN	ADAMTSL2	NRG1	MASP1
BGN	CLEC1B	FREM2	GPC4	CXCL12
PARM1	EPYC	FMOD	SDC1	HAPLN1
BMPER	CCL7	GREM1	FGF18	COL14A1
IGFBP4	SERPINB11	SMOC2	GDF5	FNDC1
SULF1	WNT2	F7	CLEC12B	GDF10
MMP15	ANGPTL2	FGF20	LGI2	ITIH5
PRG4	VWC2L	CHRDL2	IGF1	FGF9
EMILIN2	FGF22	MEGF6	C1QTNF2	FAM20A
C1QTNF5	FGL2	MMP24	COL8A2	FGF10
SERPINB5	COL11A1	SPP1	ANXA8L1	NGF
SERPINI1	SFTA2	KAZALD1	PCSK6	ITIH3
MUC19	WNT8B	S100P	IGFBP5	MMP3
SCUBE3	CCL5	CST1	NTF3	ELN
FNDC7	TNFSF14	COL17A1	WNT4	BMP4
CX3CL1	OSM	TGFB3	HPSE	FLG
ADAM20	ANGPTL1	SLIT3	MFAP5	
MMP13	HHIP	TNFSF8	GDF6	
ADAMTS17	IL17C	IL6	CLEC3B	
LAMA4	SLIT2	THBS1	VIT	
WNT3	S100A1	SFRP2	S100B	
FRAS1	FGF2	PIK3IP1	AGT	
BDNF	OGN	ACAN	SBSPON	
IGFBP7	MMP9	NTN4	ANGPT4	
TIMP3	CCL26	LGALS9	SEMA3E	
LAMC2	GPC3	MFAP4	PDGFD	
SERPINA1	EGFL7	MATN2	SERPINE2	
KY	ANXA9	WFIKKN2	EGF	
GPC1	A2M	TNFSF18	COLEC10	
COL9A3	COL25A1	MMP1	CCL2	
CD209	SEMA6D	EDIL3	ASPN	
ST14	GDF15	TNFSF10	MMP12	
CLEC14A	S100A9	CHADL	COCH	

Supplementary Table S3

Supplementary Table S4: List of genes from the inflammatory signature up-regulated in Detox-iCAF

List of the 25 genes from the inflammatory signature, which are up-regulated in Detox-iCAF cultured in collagen-coated dishes compared to ECM-myCAF maintained in plastic-dishes.

Inflammatory gene signature_up-regulated in collagen condition
CXCL3
IL24
CXCL2
ACKR1
CXCL5
CXCL8
CCL3
CCL20
CCL3L1
CCL4L2
CCL4
IL1B
CXCL1
PITPNM3
IL36RN
CXCR1
IL13
ACKR2
CXCR4
IL11
CCL28
IL36B
IL1F10
CCL24
CXCR3

Supplementary Table S4
