# Supplementary material for: Residual ANTXR1+ myofibroblasts after chemotherapy inhibit anti-tumor immunity via YAP1 signaling pathway
Source: Nat Commun. 2024 Feb 12;15:1312. doi: 10.1038/s41467-024-45595-3 (PMC10861537; doi:10.1038/s41467-024-45595-3)
Supplement: Supplementary file 3 — Reporting Summary [file 41467_2024_45595_MOESM3_ESM.pdf]

nature portfolio

Corresponding author(s): Fatima Mechta-Grigoriou and Geraldine Gentric

Last updated by author(s): 2023/01/23

Reporting Summary

Nature Portfolio wishes to improve the reproducibility of the work that we publish. This form provides structure for consistency and transparency in reporting. For further information on Nature Portfolio policies, see our Editorial Policies and the Editorial Policy Checklist.

Statistics

For all statistical analyses, confirm that the following items are present in the figure legend, table legend, main text, or Methods section.

n/a	Confirmed	
☐	☒	The exact sample size (n) for each experimental group/condition, given as a discrete number and unit of measurement
☐	☒	A statement on whether measurements were taken from distinct samples or whether the same sample was measured repeatedly
☐	☒	The statistical test(s) used AND whether they are one- or two-sided<br>*Only common tests should be described solely by name; describe more complex techniques in the Methods section.*
☐	☒	A description of all covariates tested
☐	☒	A description of any assumptions or corrections, such as tests of normality and adjustment for multiple comparisons
☐	☒	A full description of the statistical parameters including central tendency (e.g. means) or other basic estimates (e.g. regression coefficient) AND variation (e.g. standard deviation) or associated estimates of uncertainty (e.g. confidence intervals)
☐	☒	For null hypothesis testing, the test statistic (e.g. F, t, r) with confidence intervals, effect sizes, degrees of freedom and P value noted<br>*Give P values as exact values whenever suitable.*
☐	☒	For Bayesian analysis, information on the choice of priors and Markov chain Monte Carlo settings
☒	☐	For hierarchical and complex designs, identification of the appropriate level for tests and full reporting of outcomes
☒	☐	Estimates of effect sizes (e.g. Cohen's d, Pearson's r), indicating how they were calculated

Our web collection on statistics for biologists contains articles on many of the points above.

Software and code

Policy information about availability of computer code

Data collection	ScRNA-Seq data were obtained using Chromium system (10X Genomics)<br>Spatial transcriptomic data were obtained using Visium Spatial Gene Expression assay (10X Genomics)
Data analysis	Processing and analysis of scRNA-Seq data involved the use of Cell Ranger software (versions 3.1.0 and 6.0.0),the following public R packages: Seurat (https://github.com/satijalab/seurat, version 4.0.5), FastMNN (https://github.com/satijalab/seurat-wrappers), DoRothEA (https://github.com/saezlab/dorothea) and public pipeline cNMF (https://github.com/dylkot/cNMF)<br>Processing and analysis of bulk RNA-Seq was performed using public nextflow pipeline developped at Institut Curie (https://github.com/nf-core/rnaseq). Deconvolution of bulk RNA-Seq was done with BayesPrism (https://github.com/Danko-Lab/BayesPrism)<br>Processing and analysis of spatial transcriptomic data involved the use of Space Ranger software (v1.2.2), Seurat R package and cell2location (https://cell2location.readthedocs.io/en/latest/)<br><br>Statistical analysis was performed using R environment (https://cran.r-project.org) and GraphPad Prism software<br><br>Codes used for this study are available on Figshare under the https://doi.org/10.6084/m9.figshare.24271369 and on Zenodo under the https://doi.org/10.5281/zenodo.10555644

For manuscripts utilizing custom algorithms or software that are central to the research but not yet described in published literature, software must be made available to editors and reviewers. We strongly encourage code deposition in a community repository (e.g. GitHub). See the Nature Portfolio guidelines for submitting code & software for further information.

Data

Policy information about availability of data

All manuscripts must include a data availability statement. This statement should provide the following information, where applicable:

- Accession codes, unique identifiers, or web links for publicly available datasets
- A description of any restrictions on data availability
- For clinical datasets or third party data, please ensure that the statement adheres to our policy

Raw sequencing data (Fastq files from scRNAseq, spatial transcriptomic data and bulk RNAseq for CAF in culture) are available from the European Genome-Phenome Archive platform (https://ega-archive.org) under controlled access : EGAS50000000136. The controlled access is required as raw data contain identifying patient information. Data access can be granted via the EGA with completion of an institute data transfer agreement. In addition, bulk RNA sequencing data from the Retrospective Scandare Cohort 2 are available under the controlled EGA number: EGAS50000000145.

The processed data generated in this study have been deposited in the Figshare database under the following links:
-scRNAseq: https://doi.org/10.6084/m9.figshare.22147166
-Spatial transcriptomic: https://doi.org/10.6084/m9.figshare.22147103
-bulk RNAseq for CAF in culture: http://doi.org/10.6084/m9.figshare.25047746

Reference genome used: the GRCh38 reference genome dataset was used for sequence alignments.
All detailed informations are available in Methods section.

Codes used for this study are available on Figshare under the https://doi.org/10.6084/m9.figshare.24271369 and on Zenodo under the https://doi.org/10.5281/zenodo.10555644.
Data used to generate the graphs in figures are available on Figshare under the https://doi.org/10.6084/m9.figshare.24800064.

Research involving human participants, their data, or biological material

Policy information about studies with human participants or human data. See also policy information about sex, gender (identity/presentation), and sexual orientation and race, ethnicity and racism.

Reporting on sex and gender	All patients suffering from HGSOC are women.
Reporting on race, ethnicity, or other socially relevant groupings	Race, ethnicity, or other socially relevant groupings were not considered in the study design.
Population characteristics	Detailed description of retrospective and prospective cohorts of HGSOC patients are available in Tables 1 and 2 and in Methods section.
Recruitment	HGSOC patients were included prospectively in our study. No participant selection was applied in this study, all patients diagnosed with HGSOC and with available biological samples were included in this study.<br><br>All fresh samples were collected by a referent pathologist. The surgical residues, available after histopathological analyses and used in our manuscript, were not required for diagnosis. There was no interference with clinical practice. All patients treated at Institut Curie were informed orally and through an informative flyer, that their biological samples, collected through standard clinical practice, could be used for research purposes, and that by not opposing this use, they accept it.<br><br>Clinical features of prospective and retrospective cohorts are listed in Tables 1-2 and in Methods section
Ethics oversight	For both retrospective and prospective cohorts of patients, there was no interference with standard clinical practice. Samples were available for research use and not needed for diagnosis. Analysis of tumor samples was performed according to the relevant national law providing protection to people taking part in biomedical research. All patients included in the retrospective cohort were diagnosed before 2014. At that time, the principle of non-opposition was the French legal requirement. In line with this, all patients treated at Institut Curie were informed orally and through an informative flyer, that their biological samples, collected through standard clinical practice, could be used for research purposes, and that by not opposing this use, they accept it. A certificate attesting that patients have read the booklet is signed by each patient and included in her medical record. Patients' refusal, expressed either orally or written, was considered and excluded from our study. For the prospective cohorts of patients taking in charge from 2017/2018, we aligned ourselves with the General Data Protection regulation (GDPR) when it was voted in France in 2019, Institut Curie provides a systematic explicit consent form that every patient needs to sign before the samples are used for research. Then, all HGSOC patients included in prospective cohorts in our study signed this consent. The Biological Resource Centre (BRC) is integrated to the Pathology Department headed by Dr. A. Vincent-Salomon. BRC is authorized to store and manage human biological samples according to French legislation. The BRC has declared defined sample collections that are continuously incremented as and when patient consent forms are obtained (AC-2021-4366 authorization number from the French Health Ministry). The BRC follows all currently required national and international ethical rules, including the declaration of Helsinki. The BRC has also been accredited with the AFNOR NFS-96-900 quality label. In addition, the BRC collections have been declared to the CNIL (Approval Nb: 1487390 delivered February 28th, 2011). All data collected were made pseudo-anonymous for further analyses and privacy was therefore protected. Finally, the Institutional Review Board and Ethics committee of the Institute Curie Hospital Group approved all analyses realized in this study (Approval of the Tumour Micro-environment project given February 12th, 2014), as well as the National Commission for Data Processing and Liberties (Approval Nb: 1674356 delivered on March 30, 2013),

authorizations obtained by Dr. F. Mechta-Grigoriou. Finally, the Committee for the Protection of persons (CPP) expressed a favorable opinion to Dr. F. Mechta-Grigoriou's studies on tumor heterogeneity and plasticity (Approval Nb: ID-RCB: 2020-A00048-31, November 3rd, 2020).

Note that full information on the approval of the study protocol must also be provided in the manuscript.

Field-specific reporting

Please select the one below that is the best fit for your research. If you are not sure, read the appropriate sections before making your selection.

- [x] Life sciences
- [ ] Behavioural & social sciences
- [ ] Ecological, evolutionary & environmental sciences

For a reference copy of the document with all sections, see nature.com/documents/nr-reporting-summary-flat.pdf

Life sciences study design

All studies must disclose on these points even when the disclosure is negative.

Sample size

No sample size calculations were performed. No statistical method was used to predetermine sample size. Samples were collected until the sample size was sufficient to give comparison and reliable estimates.

For functional analyses (FACS, co-culture) , we analyzed at least n = 3 biological replicates.
For scRNAseq we analyzed n > 5000 cells

Data exclusions

We excluded from scRNAseq analysis cells based on pre-established quality control metrics (detailed in Methods section).
For FACS analyses, data were excluded when the tumor sample was CAF free or too small (unsufficient number of dissociated cells) to characterize both CAF and immune content.

Replication

We generated several biological replicates when it was possible. No replicates were generated for primary patient specimens.

For FACS analyses, we analyzed n = 20 primary tumors, 16 were characterized at the meanwhile for the immune content.

For functional analysis in vitro (CAF and CD8 co-culture, migration) we analyzed:
co-culture: n = 5 independent experiments, 3 CAF-S1 primary cell lines, 3 PBMC donors => 2 CAF-S1 primary cells were analyzed with two different PBMC donors each, and one CAF-S1 primary cell line was analyzed with one PBMC donor
migration: n = 8 independent experiments, 3 CAF-S1 primary cell lines, 5 PBMC donors => 2 CAF-S1 primary cells were analyzed with three different PBMC donors each, and one CAF-S1 primary cell line was analyzed with two PBMC donors
n > 5 independent experiments (3 CAF-S1 primary cell lines, 7 PBMC donors)

The number of replicates generated for in vitro studies are provided in the respective Figure legends.

Randomization

No randomization was performed, as this was not a case-control study.

Blinding

The evaluation of histological scores (H-scores) of all CAF markers and the quantifications of immune cells were carried out in a non-blinded manner by two independent researchers, including a pathologist. All quantifications gave very consistent results regardless of the person.
As there was no therapeutic intervention in this study, no blinding was performed.

Reporting for specific materials, systems and methods

We require information from authors about some types of materials, experimental systems and methods used in many studies. Here, indicate whether each material, system or method listed is relevant to your study. If you are not sure if a list item applies to your research, read the appropriate section before selecting a response.

Materials & experimental systems

n/a	Involved in the study	
[ ]	[x]	Antibodies
[ ]	[x]	Eukaryotic cell lines
[x]	[ ]	Palaeontology and archaeology
[x]	[ ]	Animals and other organisms
[ ]	[x]	Clinical data
[x]	[ ]	Dual use research of concern
[x]	[ ]	Plants

Methods

n/a	Involved in the study	
[x]	[ ]	ChIP-seq
[ ]	[x]	Flow cytometry
[x]	[ ]	MRI-based neuroimaging

Antibodies

Antibodies used

All the dilutions used in this study are listed in Supplementary Table S1

For IHC:
FAP-Rat IgG2a Vitatex, #MABS1001
CD29-Mouse IgG1 Abcam, #ab3167
SMA-Mouse IgG1 Dako, #M0851
FSP1-Rabbit IgG Abcam, #ab27957
YAP1-Rabbit IgG Cell Signaling, #14074
EPCAM-Mouse IgG1 Dako, #M0804
CD3-Mouse IgG1 DAKO, #M7254
CD8-Mouse IgG1 DAKO, #M7103
FOXP3-Mouse IgG1 Abcam, #ab20034
ANTXR1-Rabbit IgG Abcam #ab241067
Rabbit IgG-Isotype control for YAP1 Abcam, #ab171870
Rat IgG2a-Isotype control for FAP Affymetrix, #14-4321
Mouse IgG1-Isotype control for SMA Abcam, #ab91353
Mouse IgG1-Isotype control for CD29 Abcam, #ab91353
Mouse IgG1-Isotype control for FOXP3 Abcam, #ab91353
Mouse IgG1-Isotype control for CD3 Dako, #X093101
Mouse IgG1-Isotype control for CD8 Dako, #X093101
Mouse IgG1-Isotype control for EPCAM Dako, #X093101
Rabbit IgG-Isotype Control for FSP1 Dako, #X090302

For Mutliplex:
ANTXR1-Rabbit IgG, Abcam #ab246321
CD8 (AMC908), RareCyte, #52-1048-501
panCK (AE1/AE3/C11), RareCyte, #52-1015-801

For Flow-cytometry:
Brilliant Violet 605-CD326 (EpCAM) BioLegend, #324224
PE/Cy7-CD31 BioLegend, #303118
APC/Cy7-CD45 BD Biosciences, #BD-557833
PerCP5,5-CD235a Biolegend, #349110
Unconjugated-FAP R&D Systems, #MAB3715
Alexa700-CD29 BioLegend, #303020
Alexa594-αSMA R&D Systems, #IC1420T-025
PE anti human S100A4 (FSP1) BioLegend, # 370004
Fluorescent dye Zenon APC Mouse IgG1 labeling kit Thermo Fisher Scientific, #Z25051
Mouse IgG1 isotype control FAP R&D Systems, #MAB002
Alexa Fluor® 700 Mouse IgG1, κ- Isotype control CD29 BioLegend, #400144
Mouse IgG2A Alexa Fluor 594-conjugated-Isotype control αSMA R&D Systems, #IC003T
PE Mouse IgG1, κ- Isotype control FSP1 BioLegend, #400140
APC, human-CD4 Miltenyi Biotec #130-113-210
APC-Cy7-CD45 BD Biosciences, #557833
Alexa Fluor 700-CD3 BD Biosciences, #557943
PE Alexa 610-CD8 Thermofisher, #MHCD0822
Live/Dead Fixable Violet Dead Cell Stain Kit, for 405 nm excitation Thermofisher, #L34955
Unconjugated-FAP R&D Systems, #MAB3715
Alexa700-CD29 BioLegend, #303020
Mouse anti TEM8/ANTXR1 AF405 Novus Biologicals, #NB100-56585AF405
Mouse IgG1 isotype control FAP R&D Systems, #MAB002
Alexa Fluor® 700 Mouse IgG1, κ- Isotype control CD29 BioLegend, #400144
Mouse IgG1 Isotype Control AF405 Novus Biologicals, #IC003T
PE anti human EPCAM Biolegend #324205
PEcy7 anti human CD31 Biolegend #303118
APCcy7 anti human CD45 BD Biosciences #557833
FITC anti human CD235a Biolegend #349104
Unconjugated-FAP R&D Systems, #MAB3715
Alexa700-CD29 BioLegend, #303020
Mouse IgG1 isotype control FAP R&D Systems, #MAB002
Alexa Fluor® 700 Mouse IgG1, κ- Isotype control CD29 BioLegend, #400144
Alexa Fluor 700-CD3 BD Biosciences, #557943
BV510 Mouse Anti-Human CD8 BD Biosciences, #563919
Alexa Fluor® 488 Mouse Anti-Human Perforin "BD Biosciences, #563764
PE Mouse Anti-Human Granzyme B BD Biosciences, #561142

BV421 Mouse Anti-Human CD279 (PD-1) BD Biosciences, #562516
BV786 Mouse ANti-Human IFN-g BD Biosciences #563731
Fixable viability stain 780 BD Bioscience #565388
Alexa Fluor® 488 Mouse IgG2b, κ Isotype Control Perforin BD Biosciences, #558716
PE Mouse IgG1, κ Isotype Control Granzyme B BD Biosciences, #555749
BV421 Mouse IgG1, k Isotype Control PD-1 BD Biosciences, #562438
BV786 Mouse IgG1, k Isotype Control IFN-g BD Biosciences #563330

For western blot
YAP (D8H1X), Cell Signaling #14074
CYR61/CCN1, Novusbio #NB100-356
Actin, Sigma #A5441

Validation

All antibodies used in this study are commercially available. Antibodies used in a specific species or application have been appropriately validated by manufacturers for that application and this information is provided on their website and product information datasheets (see below).

FAP-Rat IgG2a Vitatex, #MABS1001 https://vitatex.com/products/mabd8-against-seprase-fap
CD29-Mouse IgG1 Abcam, #ab3167
SMA-Mouse IgG1 Dako, #M0851 https://www.agilent.com/store/productDetail.jsp?catalogId=M085101-2
FSP1-Rabbit IgG Abcam, #ab27957
YAP1-Rabbit IgG Cell Signaling, #14074 https://www.cellsignal.com/products/primary-antibodies/yap-d8h1x-xp-rabbit-mab/14074
EPCAM-Mouse IgG1 Dako, #M0804 https://www.agilent.com/store/productDetail.jsp?catalogId=M080401-2
CD3-Mouse IgG1 DAKO, #M7254 https://www.agilent.com/store/productDetail.jsp?catalogId=M725401-2
CD8-Mouse IgG1 DAKO, #M7103 https://www.agilent.com/store/productDetail.jsp?catalogId=M710301-2
FOXP3-Mouse IgG1 Abcam, #ab20034 https://www.abcam.com/products/primary-antibodies/foxp3-antibody-236ae7-ab20034.html
ANTXR1-Rabbit IgG Abcam #ab241067 https://www.abcam.com/products/primary-antibodies/tem8atr-antibody-epnci-r173-37-ab241067.html

IgG control references:
Rabbit IgG-Isotype control for YAP1 Abcam, #ab171870 https://www.abcam.com/products/primary-antibodies/rabbit-igg-polyclonal-isotype-control-chip-grade-ab171870.html
Rat IgG2a-Isotype control for FAP Affymetrix, #14-4321 https://www.thermofisher.com/antibody/product/Rat-IgG2a-kappa-clone-eBR2a-Isotype-Control/14-4321-82
Mouse IgG1-Isotype control for SMA Abcam, #ab91353 https://www.abcam.com/products/primary-antibodies/mouse-igg1-kappa-monoclonal-b116-isotype-control-ab91353.html
Mouse IgG1-Isotype control for CD29 Abcam, #ab91353 https://www.abcam.com/products/primary-antibodies/mouse-igg1-kappa-monoclonal-b116-isotype-control-ab91353.html
Mouse IgG1-Isotype control for FOXP3 Abcam, #ab91353 https://www.abcam.com/products/primary-antibodies/mouse-igg1-kappa-monoclonal-b116-isotype-control-ab91353.html
Mouse IgG1-Isotype control for CD3 Dako, #X093101 https://www.agilent.com/store/productDetail.jsp?catalogId=X093101-2
Mouse IgG1-Isotype control for CD8 Dako, #X093101 https://www.agilent.com/store/productDetail.jsp?catalogId=X093101-2
Mouse IgG1-Isotype control for EPCAM Dako, #X093101 https://www.agilent.com/store/productDetail.jsp?catalogId=X093101-2
Rabbit IgG-Isotype Control for FSP1 Dako, #X090302 https://www.agilent.com/store/productDetail.jsp?catalogId=X090302-8
ANTXR1-Rabbit IgG, Abcam #ab246321 https://www.abcam.com/products/primary-antibodies/tem8atr-antibody-epnci-r173-37-bsa-and-azide-free-ab246321.html

Flow Cytometry (FACS):
Antibody pool for CAF subset characterization from HGSOC Reference
Brilliant Violet 605-CD326 (EpCAM) BioLegend, #324224 https://www.biolegend.com/en-us/products/brilliant-violet-605-anti-human-cd326-epcam-antibody-8886
PE/Cy7-CD31 BioLegend, #303118 https://www.biolegend.com/en-us/products/pe-cyanine7-anti-human-cd31-antibody-6124
APC/Cy7-CD45 BD Biosciences, #BD-557833 https://www.bdbiosciences.com/en-fr/products/reagents/flow-cytometry-reagents/research-reagents/single-color-antibodies-ruo/apc-cy-7-mouse-anti-human-cd45.557833
PerCP5,5-CD235a Biolegend, #349110 https://www.biolegend.com/en-us/products/percp-cyanine5-5-anti-human-cd235a-glycophorin-a-antibody-9002
Unconjugated-FAP R&D Systems, #MAB3715 https://www.rndsystems.com/products/human-fibroblast-activation-protein-alpha-fap-antibody-427819_mab3715
Alexa700-CD29 BioLegend, #303020 https://www.biolegend.com/en-us/products/alexa-fluor-700-anti-human-cd29-antibody-3420
Alexa594-αSMA R&D Systems, #IC1420T-025 https://www.rndsystems.com/products/human-alpha-smooth-muscle-actin-alexa-fluor-594-conjugated-antibody-1a4_ic1420t
PE anti human S100A4 (FSP1) BioLegend, # 370004 https://www.biolegend.com/en-us/products/pe-anti-human-s100a4-antibody-13082
Fluorescent dye Zenon APC Mouse IgG1 labeling kit Thermo Fisher Scientific, #Z25051 https://www.thermofisher.com/order/catalog/product/fr/en/Z25051
IgG controls Reference
Mouse IgG1 isotype control FAP R&D Systems, #MAB002 https://www.rndsystems.com/products/mouse-igg-1-isotype-control_mab002
Alexa Fluor® 700 Mouse IgG1, κ- Isotype control CD29 BioLegend, #400144 https://www.biolegend.com/en-us/products/alexa-

fluor-700-mouse-igg1-kappa-isotype-ctrl-3376
Mouse IgG2A Alexa Fluor 594-conjugated-Isotype control αSMA R&D Systems, #IC003T https://www.rndsystems.com/products/mouse-igg2a-alexa-fluor-594-conjugated-isotype-control_ic003t
PE Mouse IgG1, κ- Isotype control FSP1 BioLegend, #400140 https://www.biolegend.com/en-us/products/pe-mouse-igg1-kappa-isotype-ctrl-icfc-3032
Antibody pool for T lymphocytes characterization from HGSOC Reference
APC, human-CD4 Miltenyi Biotec #130-113-210 https://www.miltenyibiotec.com/FR-en/products/cd4-antibody-anti-human-vit4.html#apc:100-tests-in-200-ul
APC-Cy7-CD45 BD Biosciences, #557833 https://www.bdbiosciences.com/en-fr/products/reagents/flow-cytometry-reagents/research-reagents/single-color-antibodies-ruo/apc-cy-7-mouse-anti-human-cd45.557833
Alexa Fluor 700-CD3 BD Biosciences, #557943 https://www.bdbiosciences.com/en-fr/products/reagents/flow-cytometry-reagents/research-reagents/single-color-antibodies-ruo/alexa-fluor-700-mouse-anti-human-cd3.557943
PE Alexa 610-CD8 Thermofisher, #MHCD0822 https://www.thermofisher.com/antibody/product/CD8-Antibody-clone-3B5-Monoclonal/MHCD0822
Live/Dead Fixable Violet Dead Cell Stain Kit, for 405 nm excitation Thermofisher, #L34955 https://www.thermofisher.com/order/catalog/product/L34955?ef_id=Cj0KCQiAgaGgBhC8ARIsAAAyLfHgxTgwb7b_OtonFTQvMuV8wv7QcgKn2AYiuAO9bhjgabKu2plytYAaAqc5EALw_wcB:G:s&s_kwcid=AL!3652!3!601175229291!!!g!!!17329199729!133905464141&cid=bid_pca_frg_r01_co_cp1359_pjt0000_bid00000_0se_gaw_dy_pur_con&gclid=Cj0KCQiAgaGgBhC8ARIsAAAyLfHgxTgwb7b_OtonFTQvMuV8wv7QcgKn2AYiuAO9bhjgabKu2plytYAaAqc5EALw_wcB
Antibody pool for CAF-S1 primary cell characterization Reference
Unconjugated-FAP R&D Systems, #MAB3715 https://www.rndsystems.com/products/human-fibroblast-activation-protein-alpha-fap-antibody-427819_mab3715
Alexa700-CD29 BioLegend, #303020 https://www.biolegend.com/en-us/products/alexa-fluor-700-anti-human-cd29-antibody-3420
Mouse anti TEM8/ANTXR1 AF405 Novus Biologicals, #NB100-56585AF405 https://www.novusbio.com/products/tem8-antxr1-antibody-200c1339-sb20-_nb100-56585af405
IgG controls Reference
Mouse IgG1 isotype control FAP R&D Systems, #MAB002 https://www.rndsystems.com/products/mouse-igg-1-isotype-control_mab002
Alexa Fluor® 700 Mouse IgG1, κ- Isotype control CD29 BioLegend, #400144 https://www.biolegend.com/en-us/products/alexa-fluor-700-mouse-igg1-kappa-isotype-ctrl-3376
Mouse IgG1 Isotype Control AF405 Novus Biologicals, #IC003T https://www.novusbio.com/products/igg2a-isotype-control-20102_ic003t
Antibody pool for CAF-S1 cell sorting Reference
PE anti human EPCAM Biolegend #324205 https://www.biolegend.com/en-us/products/pe-anti-human-cd326-epcam-antibody-3757
PEcy7 anti human CD31 Biolegend #303118 https://www.biolegend.com/en-us/products/pe-cyanine7-anti-human-cd31-antibody-6124
APCcy7 anti human CD45 BD Biosciences #557833 https://www.bdbiosciences.com/en-fr/products/reagents/flow-cytometry-reagents/research-reagents/single-color-antibodies-ruo/apc-cy-7-mouse-anti-human-cd45.557833
FITC anti human CD235a Biolegend #349104 https://www.biolegend.com/en-us/products/fitc-anti-human-cd235a-glycophorin-a-antibody-6701
Unconjugated-FAP R&D Systems, #MAB3715 https://www.rndsystems.com/products/human-fibroblast-activation-protein-alpha-fap-antibody-427819_mab3715
Alexa700-CD29 BioLegend, #303020 https://www.biolegend.com/en-us/products/alexa-fluor-700-anti-human-cd29-antibody-3420
IgG controls Reference
Mouse IgG1 isotype control FAP R&D Systems, #MAB002 https://www.rndsystems.com/products/mouse-igg-1-isotype-control_mab002
Alexa Fluor® 700 Mouse IgG1, κ- Isotype control CD29 BioLegend, #400144 https://www.biolegend.com/en-us/products/alexa-fluor-700-mouse-igg1-kappa-isotype-ctrl-3376
Antibody pool for CD8+ cell solation for functional assays Reference
Alexa Fluor 700-CD3 BD Biosciences, #557943 https://www.bdbiosciences.com/en-fr/products/reagents/flow-cytometry-reagents/research-reagents/single-color-antibodies-ruo/alexa-fluor-700-mouse-anti-human-cd3.557943
BV510 Mouse Anti-Human CD8 BD Biosciences, #563919 https://www.bdbiosciences.com/en-fr/products/reagents/flow-cytometry-reagents/research-reagents/single-color-antibodies-ruo/bv510-mouse-anti-human-cd8.563919
Alexa Fluor® 488 Mouse Anti-Human Perforin "BD Biosciences, #563764
" https://www.bdbiosciences.com/en-fr/products/reagents/flow-cytometry-reagents/research-reagents/single-color-antibodies-ruo/alexa-fluor-488-mouse-anti-human-perforin.563764
PE Mouse Anti-Human Granzyme B BD Biosciences, #561142 https://www.bdbiosciences.com/en-fr/products/reagents/flow-cytometry-reagents/research-reagents/single-color-antibodies-ruo/pe-mouse-anti-human-granzyme-b.561142
BV421 Mouse Anti-Human CD279 (PD-1) BD Biosciences, #562516 https://www.bdbiosciences.com/en-fr/products/reagents/flow-cytometry-reagents/research-reagents/single-color-antibodies-ruo/bv421-mouse-anti-human-cd279-pd-1.562516
BV786 Mouse ANti-Human IFN-g BD Biosciences #563731https://www.bdbiosciences.com/en-us/products/reagents/flow-cytometry-reagents/research-reagents/single-color-antibodies-ruo/bv786-mouse-anti-human-ifn.563731
Fixable viability stain 780 BD Bioscience #565388 https://www.bdbiosciences.com/en-fr/products/reagents/flow-cytometry-reagents/research-reagents/single-color-antibodies-ruo/fixable-viability-stain-780.565388
IgG controls Reference
Alexa Fluor® 488 Mouse IgG2b, κ Isotype Control Perforin BD Biosciences, #558716 https://www.bdbiosciences.com/en-fr/products/reagents/flow-cytometry-reagents/research-reagents/single-color-antibodies-ruo/alexa-fluor-488-mouse-igg2b-isotype-control.558716
PE Mouse IgG1, κ Isotype Control Granzyme B BD Biosciences, #555749 https://www.bdbiosciences.com/en-fr/products/reagents/flow-cytometry-reagents/research-reagents/flow-cytometry-controls-and-lysates/pe-mouse-igg1-isotype-control.555749
BV421 Mouse IgG1, k Isotype Control PD-1 BD Biosciences, #562438 https://www.bdbiosciences.com/en-fr/products/reagents/flow-

cytometry-reagents/research-reagents/flow-cytometry-controls-and-lysates/bv421-mouse-igg1-k-isotype-control.562438
BV786 Mouse IgG1, k Isotype Control IFN-g BD Biosciences #563330 https://www.bdbiosciences.com/en-us/products/reagents/flow-cytometry-reagents/research-reagents/flow-cytometry-controls-and-lysates/bv786-mouse-igg1-k-isotype-control.563330

All flow cytometry reagents are titrated on the relevant positive or negative cells.
For antibodies used in immunoblotting, specificity was validated by detection of the correct molecular weight upon siRNA and non-specific detection was minimized through optimizing concentration and blocking reagent.

For western blot
YAP (D8H1X) https://www.cellsignal.com/product/productDetail.jsp?productId=14074
CYR61/CCN1 https://www.novusbio.com/PDFs/NB100-356.pdf
Actin https://www.sigmaaldrich.com/specification-sheets/141/510/A5441-BULK________SIGMA____.pdf

Eukaryotic cell lines

Policy information about cell lines and Sex and Gender in Research

Cell line source(s)	Primary CAF cell lines were isolated from HGSOC fresh tumor samples.<br>Fresh HGSOC samples from the operating room were cut into small pieces and incubated on a plastic petri dish (Falcon, #353003) in DMEM (Gibco #11965092) supplemented with 10% FBS (Biosera, #FB-1003/500), and 1%penicillin (100 U ml−1) and streptomycin (100 µg ml−1) (Gibco #15140122) at 37°C in 5% of CO_2 and 1.5% of O_2 for at least 2 weeks to let fibroblasts spread and expand. The media was renewed 2-3 times per week. When fibroblasts reached 50% of confluency, they were detached with trypsin and plated in new petri dishes. All experiments were performed with fibroblasts until passage 10 to avoid CAF senescence.<br><br>CAOV3 ovarian tumor cell line is from American Type Culture Collection, ATCC
Authentication	Identity of primary CAF-S1 fibroblasts in culture was next validated by flow cytometry (FAP+, CD29+ and ANTXR1) and bulk-RNAseq<br><br>For CAOV3 cells, the cell line identity was verified by using the Short Tandem Repeat (STR) DNA profiling (Promega # B9510) method.
Mycoplasma contamination	All cells were mycoplasma negative
Commonly misidentified lines (See ICLAC register)	No cells used were listed in the database of misidentified cell lines

Clinical data

Policy information about clinical studies
All manuscripts should comply with the ICMJEguidelines for publication of clinical research and a completedCONSORT checklist must be included with all submissions.

Clinical trial registration	NA
Study protocol	NA
Data collection	Prospective cohort 1: All patients were included from 2017-2020<br>Prospective cohort 2: All patients were included from 2018-2021<br>Retrospective Curie 1 cohort : All patients were included from 2000-2014<br>Retrospective SCANDARE Curie 2 cohort: All patients were included from 2017-2023
Outcomes	Detailed descriptions of prospective and retrospective cohorts of breast cancer patients are available in Table 1

Plants

Seed stocks	NA
Novel plant genotypes	NA
Authentication	NA

Flow Cytometry

Plots

Confirm that:

- [ ] The axis labels state the marker and fluorochrome used (e.g. CD4-FITC).
- [x] The axis scales are clearly visible. Include numbers along axes only for bottom left plot of group (a 'group' is an analysis of identical markers).
- [x] All plots are contour plots with outliers or pseudocolor plots.
- [x] A numerical value for number of cells or percentage (with statistics) is provided.

Methodology

Sample preparation	Fresh human HGSOC primary tumors were obtained directly from the operating room and cut into small fragments by a mechanical and enzymatic digestion in CO2-independant medium (Gibco #18045-054) supplemented with 2mg/ml collagenase I (Sigma #C0130), 2mg/ml hyaluronidase (Sigma #H3506) and 25 mg/ml DNaseI (Roche #11284932001) during 45 minutes (min) at 37°C with shaking (180 rpm). After tissue digestion, cells were filtrated through a 40 µm cell strainer (Falcon #352340) and resuspended in PBS+ solution (PBS, Gibco #14190; EDTA 2mM, Gibco #15575; Human serum 1%, BioWest #S4190-100). Cells were next separated into two groups for analyzing panels of CAF subsets and immune cells, separately.<br><br>Characterization of CAF populations: Cells were stained with an antibody mix for the detection of both cell surface and intracellular staining containing anti-EPCAM-BV605 (1:50; BioLegend, #324224), anti-CD45-APCCy7 (1:20; BD Biosciences, #BD-557833), anti-CD31-PE/Cy7 (1:100; BioLegend, #303118), anti-CD235a-PerCP5.5 (1:50; Biolegend, #349110), anti-FAP-APC (1:100; R&D Systems, #MAB3715) conjugated with the fluorescent dye Zenon APC Mouse IgG1 labeling kit (1/100, ThermoFisher Scientific #Z25051), anti-CD29-Alexa700 (1:100; BioLegend, #303020) and anti-SMA-Alexa594 (1:25; R&D Systems, #IC1420T-025), anti-FSP1-PE (1:25; BioLegend, # 370004) and anti-ANTXR1-AF405 (1:25; Novus Biologicals, #NB100-56585AF405). For each CAF marker, the isotype control antibody was: iso-anti-FAP (primary antibody, 1:200, R&D Systems, #MAB002), iso-anti-CD29 (1:100, BioLegend, #400144), iso-anti-SMA (1:25, R&D Systems, #IC003T), iso-anti-FSP1 (primary antibody, 1:20, BioLegend, #400139) and iso-anti-ANTXR1 (1:25; Novus Biologicals, #IC003T).<br>For surface staining, cell suspensions were stained immediately after dissociation of samples during 15 min at RT with the antibody mix in PBS+ solution and 2.5 µg/ml DAPI (Thermo Fisher scientific, #D1306) was added just before flow cytometry analysis. For intracellular staining, cells were stained with a violet live/dead marker (1:1000, Thermofisher Scientific #L34955) for 10 min at room temperature (RT) in PBS (Gibco #14190) to exclude dead cells and then fixed in 4% paraformaldehyde (PFA) (Electron Microscopy Sciences, #15710) for 20 min at room temperature (RT). After a rapid washing step with PBS+ solution (PBS supplemented with EDTA 2mM and 1% Human serum), cells were stained with an antibody mix during 30 min at room temperature (RT). Antibodies are suspended in PBS+ solution with 0.1% of Saponin (Sigma-Aldrich #S7900) and corresponding isotype control mix antibodies are used for each experiment.<br><br>Characterization of T lymphocytes: HGSOC fresh tissues were collected and digested, as described above. Cells were stained with an antibody mix for the detection of cell surface staining containing anti-CD45-APC-Cy7 (1:20; BD Biosciences, #557833), anti-CD3-AlexaFluo700 (1:40; BD Biosciences, #557943), anti-CD8-PE/Alexa610 (1:80; Thermofisher, #MHCD0822) and anti-CD4-APC (1:20; Miltenyi Biotec, #130-113-210). Cells were stained with a violet live/dead marker (1/1000, Thermofisher Scientific #L34955) for 10 min at RT in PBS (Gibco #14190) to exclude dead cells. Cells were incubated for 20min at RT with the antibody mix in PBS+ solution.
Instrument	LSRFortessa analyzer (BD biosciences)
Software	FlowJo version 10.4.2 (LLC)
Cell population abundance	At least 5×105 events were recorded.<br>Percentage of CAF are calculated among total viable cells. Percentage of ANTXR1+ cells is calculated among the CAF-S1 population (FAP+ CD29+).<br>Percentage of CD8+ T cells is calculated among CD3+ T cells.
Gating strategy	Characterization of CAF populations: Cells were first gated based on forward (FSC-A) and side (SSC-A) scatters (measuring cell size and granularity, respectively) to exclude debris. Dead cells were excluded based on their positive staining for Live/Dead. Gating included EPCAM−, CD45−, CD31−, CD235a− cells, to remove epithelial (EPCAM+), hematopoietic (CD45+), endothelial (CD31+) and red blood cells (CD235a+). Cells from the negative fraction were next examined using CAF markers, including FAP, CD29, αSMA, FSP1 and ANTXR1.<br><br>Characterization of T lymphocytes: Cells were first gated based on forward (FSC-A) and side (SSC-A) scatters (measuring cell size and granularity, respectively) to exclude debris. Dead cells were excluded based on their positive staining for Live/Dead. Gating included CD45+ and CD3+ to gate on T cells. We used CD4+ and CD8+ to determine the % of CD4+ T cells and CD8+ T cells among CD3+ T cells.

- [x] Tick this box to confirm that a figure exemplifying the gating strategy is provided in the Supplementary Information.
